# The practice of dentistry in intensive care units in Brazil

**DOI:** 10.5935/0103-507X.20180044

**Published:** 2018

**Authors:** Davi Francisco Casa Blum, José Augusto Santos da Silva, Fernando Martins Baeder, Álvaro Della Bona

**Affiliations:** 1 Faculdade de Odontologia, Universidade de Passo Fundo - Passo Fundo (RS), Brasil.; 2 Serviço de Cirurgia Bucomaxilofacial, Fundação de Beneficência Hospital de Cirurgia - Aracajú (SE), Brasil.; 3 Universidade Cruzeiro do Sul - São Paulo (SP), Brasil.

**Keywords:** Dental health surveys, Diagnosis, oral, Oral health, Intensive care units, Brazil

## Abstract

**Objective:**

To evaluate the practice of dentistry in intensive care units.

**Methods:**

An observational survey study was conducted in which questionnaires were sent via the online platform for collaboration in intensive care research in Brazil (AMIBnet). The study was carried out from June to October 2017. The questionnaires, which contained 26 closed questions about hospitals and dentistry practices in the intensive care units, were sent to 4,569 professionals from different specialties practicing in the units.

**Results:**

In total, 203 questionnaires were returned, resulting in a response rate of 4.44%. Most of the responses were from intensive care units in the Southeast region of the country (46.8%). Public hospitals (37.9%) and private hospitals (36.4%) had similar participation rates. Of the respondents, 55% indicated that a bedside dentistry service was present, and they were provided in different ways.

**Conclusion:**

The presence of dentistry services and oral health service delivery training and protocols were correlated. The oral care methods varied greatly among the intensive care units surveyed.

## INTRODUCTION

Inpatients with poor oral health have a higher risk of unfavorable outcomes due to their increased risk of respiratory infection. It is known that the risk of poor progression as a result of respiratory infections is increased in hospitalized patients with poor oral hygiene.^(1-4)^ Recent systematic reviews point to the importance of protocols for chemical and mechanical control of oral colonization for the prevention of unfavorable systemic and oral health outcomes.^(5-8)^

Dental care in intensive care units (ICUs) is important and cost effective for the prevention and control of diseases such as respiratory infections.^(5,6,8-11)^ Oral care is perceived as highly important for patients under mechanical ventilation (MV) in the ICU by more than 90% of nursing professionals. In addition to this care being considered difficult to perform, when it is not properly taught to the team, the task becomes even more complex for those who perform it.^(12-15)^

It is important to determine the impact of oral care protocols on the health of patients. When one of these protocols is present, the quality of care activities is significantly better and the participation of the team involved in the care is fuller, evidencing the importance of the presence of these protocols.^(8,12,13,16,17)^ In a study that asked nursing professionals in ICUs in Brazilian hospitals about oral health care routines and protocols, the presence of dental surgeons in ICU care routines who implement institutional oral care protocols and train the teams was found to lead to positive and more consistent attitudes by the nursing teams regarding patients' oral health.^(18)^

Epidemiological data and data on professional practices in health care are important for the design of health policies and strategies. The Brazilian ICUs (*UTIs Brasileiras*) Project,^(19)^ an initiative of entities related to intensive care, aims to characterize the profiles of Brazilian ICUs. Through this project, data related to the ICU characteristics, hospitalizations, patient demographics and main diagnoses, use of invasive supports and main outcomes are available for use in research and the planning of health services delivery. The Association of Brazilian Intensive Medicine (*Associação de Medicina Intensiva Brasileira* - AMIB) also recently released a census of Brazilian ICUs with data from 2016. However, data related to the provision of dentistry services are not available on these platforms.^(20)^

This study aimed to evaluate the practice of dentistry in ICUs in Brazil.

## METHODS

We conducted an observational survey study by sending questionnaires via the online platform for collaboration in intensive care research in Brazil (AMIBnet). The text of the invitation sent by electronic mail noted that the confidentiality of the data regarding the respondent and his/her institution would be ensured. A positive response to the e-mail invitation was considered agreement to participate in the study.

This study was approved by the Research Committee at *Universidade de Passo Fundo* (CEP 1,879,807) and followed the ethical standards of the Declaration of Helsinki.

Professionals from different fields (medicine, nursing, dentistry, physical therapy, speech therapy, nutrition and psychology) with direct connections to professional practice in intensive therapy participated in the cooperation network. The questionnaires were sent to all the professionals in the network. Contact was made using personal e-mails and cell phone text messages to direct participants to a questionnaire hosted on the SurveyMonkey^®^ platform. The survey was conducted from June to October 2017, and the questionnaires were sent to 4,569 network professionals in four e-mail blasts (in June, July, August and September).

The questionnaire was composed of 26 questions that were mostly closed and objective. They covered information about the professional who answered the questionnaire, the hospital and ICU in which he/she worked, the performance of dentistry professionals in their ICU and information about oral care protocols practiced in the ICU.

The results were tabulated and analyzed using the Statistical Package for the Social Science (SPSS), version 20 (IBM). A descriptive statistical analysis of frequencies was used to characterize the profiles of the ICUs as well as their dental practices. An analysis of contingency tables and a chi-square test with a significance level of 5% were used to correlate some variables.

## RESULTS

We obtained a total of 203 responses; that is, the response rate was 4.44%. Of the respondents, 43.8% were medical doctors, 19.7% were nurses, 11.3% were dental surgeons, and 25.2% were from other professions.

**Figure 1:**
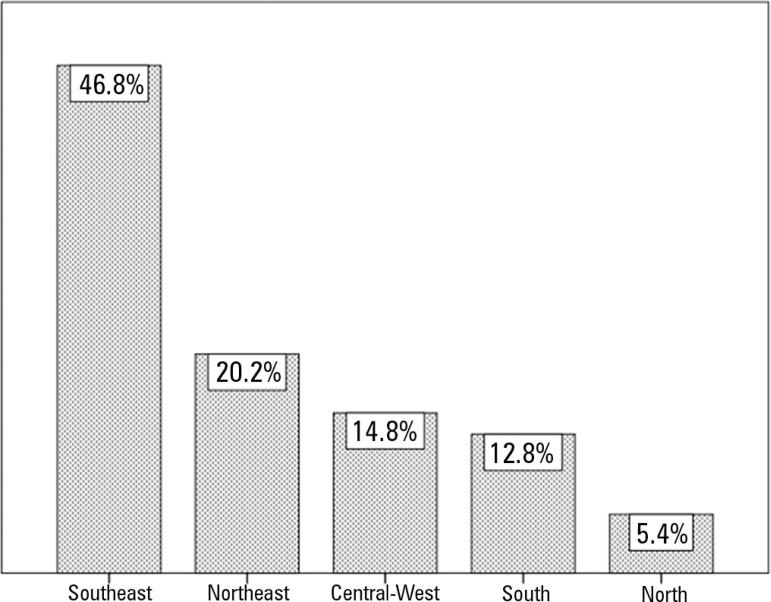
Intensive care units surveyed by Brazilian region.

**Figure 2:**
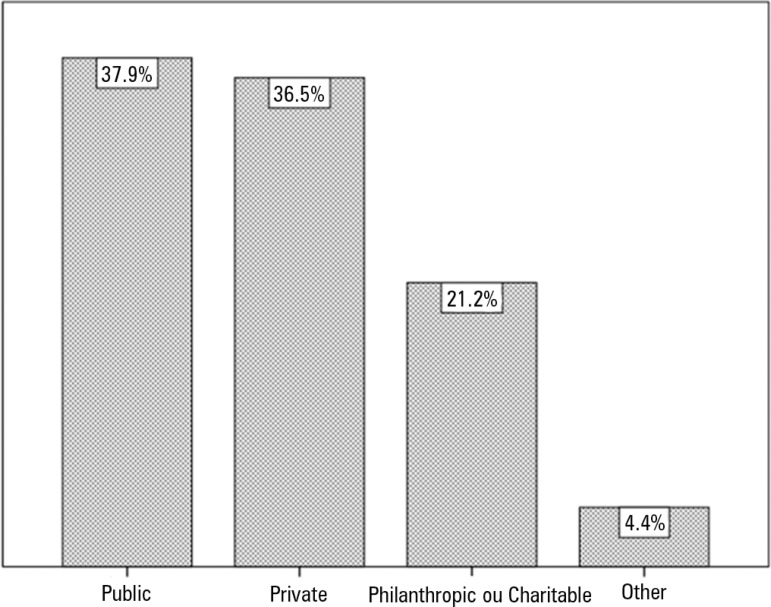
Intensive care units surveyed by type of hospital.

Most of the responses came from ICUs in the Southeast region of the country (46.8%). Public hospitals (37.9%) and private hospitals (36.4%) had similar participation rates in the results. Figures 1 and 2 illustrate and expand on the above results.

**Figure 3:**
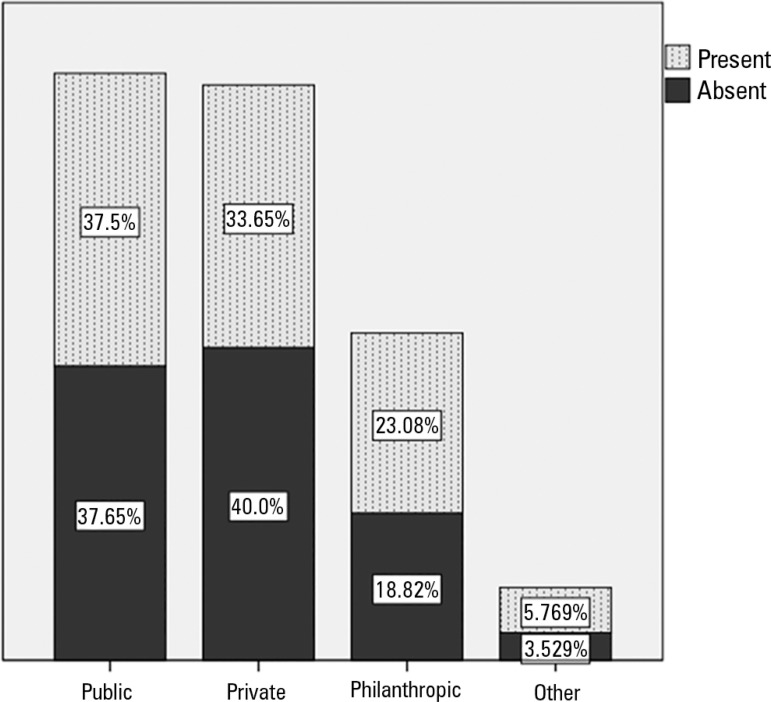
Dentistry services in intensive care units in different types of hospitals (percentages are displayed in the columns).

When asked about the availability of a hospital dentistry service (dental surgeon) at the bedside in the ICU, 55% of the respondents noted the presence of such a service. When we evaluated only the responses of physicians, nurses and managers (128 responses), the number of positive responses was 44.5%. There was no significant difference (p = 0.703) between the type of hospital and the presence of a dental surgeon in the ICU. Figure 3 illustrates these findings. The Brazilian region in which the ICU was located also did not influence the presence of a dental service (p = 0.666).

**Figure 4:**
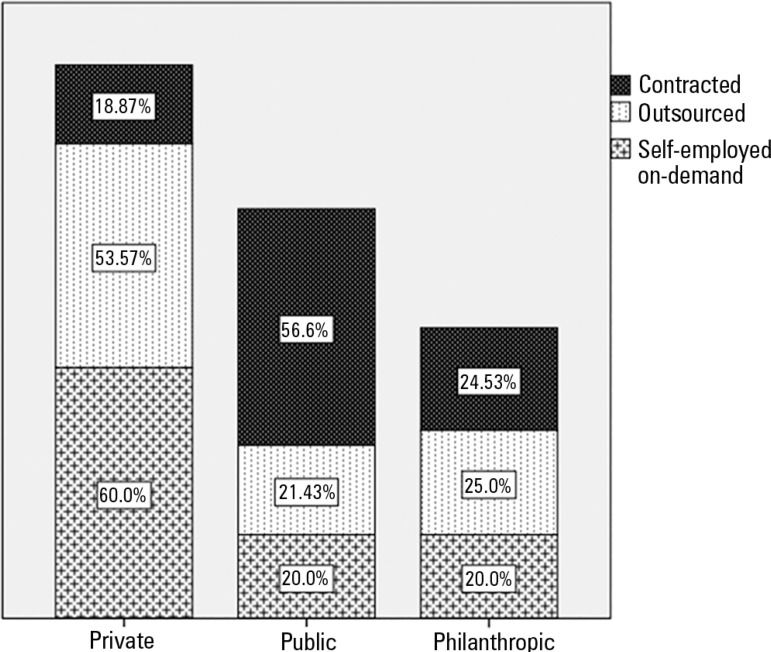
Work contracts of dental surgeons according to the type of hospital (percentages are displayed in the columns).

Regarding the dental surgeons' work regimes in the ICUs, the majority (57.4%) were hired by the hospital; 27.7% were outsourced and 14.9% were self-employed professionals who provided services on demand. We observed a significant difference (p = 0.002) between the types of hospitals and the work arrangements of their dental surgeons: public hospitals tended to hire professionals, whereas in private hospitals, most dentists were outsourced or self-employed on-demand professionals. Figure 4 illustrates and expands on these data.

Of the dental professionals who worked in ICUs, typically (52.5%) only one professional was responsible for caring for the patients, while in 30.7% of the cases, a team of three or more professionals were present. Normally (46.5%), dentists worked less than 10 hours per week in ICUs, while schedules of 10 - 20 hours and 20 - 40 hours per week each accounted for 24.8% of the remaining cases. Approximately 69% of dentists working in ICUs participated, at least occasionally, in multidisciplinary rounds and were members of multiprofessional teams.

Most of the ICUs (68.4%) received regular training regarding the oral hygiene and health care of patients, and a defined oral care protocol was present in 73.4% of the ICUs surveyed. The presence of a hospital dental service had a significant relationship with both situations: training (p = 0.004) and the presence of care protocols (p = 0.001). We also observed a relationship between the presence of protocols and regular training (p < 0.001).

**Figure 5:**
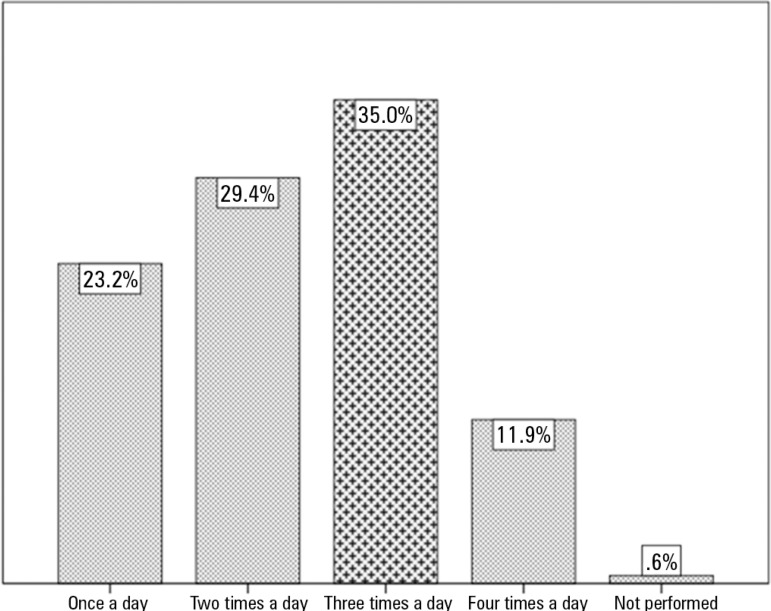
Frequency of oral hygiene in intensive care unit patients.

Only one ICU surveyed reported not performing oral hygiene on inpatients. The frequency of providing oral hygiene in ICUs varied greatly, and figure 5 illustrates and expands on these findings. Oral hygiene was the responsibility of nursing technicians in 75.7% of the cases and of the dental surgeon in 13%, with other professionals (nurses) or more than one professional involved in the other cases.

The products used in oral hygiene also varied. Oral chlorhexidine (aqueous 0.12 - 0.2%) was used in 80.8% of the ICUs surveyed. Toothbrushes were used for all inpatients in 42.2% of the ICUs surveyed. In 37.2% of the ICUs, toothbrushes were used only for conscious patients and/or patients able to perform their own hygiene, and in 20.3% of the ICUs, toothbrushes were not used. When used, toothbrushes were disposable in 23.7% of the cases, disinfected with an antiseptic in 22.6% and washed only with water in 37.3%; no disinfection was performed in 16.4%.

## DISCUSSION

We obtained 203 completed questionnaires in our study for a response rate of 4.4% out of the total of 4,569 professionals contacted in a country where, according to the 2016 census, there were 1,961 ICUs.^(20)^ In this case, the 203 responses from ICUs, considering that a few responses were from the same hospital, represent a percentage of more than 4.4% of Brazilian ICUs.

Regarding the distribution of ICUs, we obtained results consistent with the reality in Brazil,^(20)^ as most of the ICUs are concentrated in the Southeast region (50% in the national census *versus* 46.8% in our study), followed by the Northeast (20.2% *versus* 20.2%), South (14.7% *versus* 12.8%), Central-West (8.46% *versus* 14.8%) and North (6.6% *versus* 5.4%) regions. The type of hospital contacted also reflected the Brazilian reality; however, in our study, we had a higher rate of responses from public hospitals (37.9% *versus* 28% in the Brazilian census) despite private hospitals being present in greater numbers in Brazil (44% in the census *versus* 36.4% in our study). We believe that this small difference did not affect our results, considering the lack of relationship between the type of hospital and most of the variables surveyed.

In studies with questionnaires, there is a respondent interest bias. As our study focused on the subject of dental care, we obtained more answers from dental surgeons (11.3%) than the actual proportion of these professionals among those who received the questionnaires. To assess this potential bias, we analyzed the question of the presence of a bedside dentistry service considering only the responses of doctors, nurses and managers and obtained a reduction from 55% to 44.5% in the number of responses affirming the presence of this service - a proportion that should be more similar to the Brazilian reality.

These data are lower than expected, considering that since 2010, Resolution No. 7 of the Board of Directors of the National Health Surveillance Agency (*Agência Nacional de Vigilância Sanitária* - ANVISA), which establishes the minimum requirements for the operation of ICUs in Brazil, has required, in Article 18, the compulsory presence of bedside dental care provided by in-house or outsourced professionals.

The type of hospital (public, private or philanthropic) did not influence the presence of a dentistry service in the ICUs, which may suggest that private hospitals have no greater interest in improving the quality of services in this area. Public hospitals tend to hire dental professionals, while in private hospitals, most of the professionals are outsourced or self-employed on-demand professionals. Such staffing is probably due to the trend towards cost containment in private hospitals and to public policies for formal staffing.

The oral care protocols varied among institutions. Usually, protocols are related to the prevention of nosocomial pneumonia.^(8,12,14,21)^ The presence of protocols varies depending on the study, with a study in Croatia finding a rate of 65%^(14)^ and one in the United States finding a rate of 25%.^(22)^ In a worldwide study that included 1,730 responses from 77 countries,^(9)^ only 27% of the ICUs included oral hygiene as part of their package for the prevention of ventilator-associated pneumonia (VAP) in the ICU. Our study indicates that a defined oral care protocol is present in 73.4% of Brazilian ICUs.

Studies demonstrate the importance of training and the dissemination of institutional hygiene protocols.^(14,15,17,23)^ Our study corroborates this finding and indicates that there is a correlation between the presence of a bedside dentistry service and both the use of these protocols and the performance of regular trainings.

Oral care varies by institution.^(12,14,21)^ The use of manual toothbrushes is controversial, and some studies suggest that they may not be beneficial in the prevention of VAP.^(10,24)^ Toothbrushes are used at least once a day by less than 40% of nursing professionals in ICUs in the United States; the use of some type of mouthwash was reported by 96%, and only 20% routinely used chlorhexidine-based mouthwashes.^(12)^ In a survey conducted in Switzerland, 25% of the hospitals surveyed reported having a protocol for the prevention of VAP, 75% completed oral hygiene routines for patients three times a day, 90% reported the use of toothbrushes for oral hygiene care in ICU patients, and 67% used chlorhexidine as a mouthwash.^(22)^ In our study, only one ICU (0.56%) did not perform oral hygiene care for hospitalized patients. Oral chlorhexidine was used in 80.8% of the ICUs surveyed, and toothbrushes were used in all patients in 42.2% of the units. Some institutions implemented the use of toothbrushes with reservations based on the types of patients they were used with and with care to address contamination.

## CONCLUSION

About half of Brazilian intensive care units provide some type of bedside dentistry service, although the details of these services vary. The practice of dentistry in intensive care units is irregular at the national level, and service delivery is performed in a non-standard way.

Institutions that offer bedside dentistry services tended to be more organized regarding oral health training requirements and service delivery protocols.
